# Examining the relationship of concurrent obesity and tobacco use disorder on the development of substance use disorders and psychiatric conditions: Findings from the NESARC-III

**DOI:** 10.1016/j.dadr.2023.100162

**Published:** 2023-04-23

**Authors:** L.J. Fields, W. Roberts, I. Schwing, M. McCoy, T.L. Verplaetse, M.R. Peltier, R.F. Carretta, Y. Zakiniaeiz, R. Rosenheck, S.A. McKee

**Affiliations:** a Department of Psychology, Arcadia University, 450 S Easton Rd, Glenside, PA 19038, United States; b Department of Psychiatry, Yale School of Medicine, 300 George St #901, New Haven, CT 06511, United States; c VA Connecticut Healthcare System, 950 Campbell Ave, West Haven, CT 06516, United States

**Keywords:** Obesity, Tobacco use disorder (TUD), Substance use disorder (SUD), Multimorbidity

## Abstract

**Background:**

Multimorbidity is linked to worse health outcomes than single health conditions. However, recent studies show that obesity may reduce the risk of developing substance use disorders (SUDs), particularly in vulnerable populations. We investigated how comorbid obesity and tobacco use disorder (TUD) relate to the risk of SUDs and psychiatric conditions.

**Methods:**

Data was used from 36,309 individuals who completed the National Epidemiological Survey on Alcohol and Related Conditions - Wave III. Individuals who met the DSM-5 criteria for TUD in the last year were defined as the TUD group. Obesity was defined as having a body mass index (BMI) greater than 30 kg/m^2^. Using this information, individuals were grouped into categories, with people being identified as either having obesity, TUD, both obesity and TUD, or not having either obesity or TUD (comparison). Groups were compared against their comorbid diagnoses of either an additional SUD or psychiatric conditions.

**Results:**

Controlling for demographic characteristics, we found that individuals with obesity including those individuals with TUD, had lower rates of comorbid SUD diagnosis than individuals with TUD alone. Additionally, individuals with combined TUD and obesity, and those with TUD alone, had the highest rates of comorbid psychiatric disorder diagnosis.

**Conclusions:**

The current study aligns with previous research suggesting that obesity may reduce risk of substance use disorders, even in individuals who have other risk factors promoting harmful substance use (e.g., tobacco use). These findings may inform targeted intervention strategies for this clinically relevant subpopulation.

## Highlights

• We examined rates of SUD and psychiatric disorders in people with TUD, obesity, and both health conditions.
• People with TUD and obesity have lower rates of SUD than people with TUD in the normal weight range.
• Obesity (BMI>30) is associated with lower risk of SUD.

## 1 Introduction

Multimorbidity affects up to 1/3 of all adults and 1/2 of those with preexisting chronic health conditions, resulting in increased healthcare visits, hospital stays, and costs (GBD, 2019; Han et al., 2018; Skou et al., 2022). Multimorbidity increases the risk of death, lowers quality of life, and leads to poorer health outcomes compared to having a single condition (Bhalla et al., 2018). These conditions also disproportionately affect lower socioeconomic status communities (Katikireddi et al., 2017; Salisbury, 2012; Skou et al., 2022). Additionally, individuals with multimorbidities are also 2–3 times more likely to experience depression (Fortin et al., 2004, 2006; Read et al., 2017). To improve treatment approaches, it is important to further characterize and understand multimorbidity as most healthcare systems focus on single conditions and, thus, are not equipped to handle them (Atun, 2015).

Two conditions that are of particular interest to the current study are obesity and tobacco use disorder (TUD) because of their outsized impact on both physical and mental health. Each disorder accounts for more preventable deaths, particularly in the developed world, than most other conditions combined (Powell-Wiley et al., 2021; CDC, 2010). The co-occurence of these conditions is common, impacting an estimated 3–7% of the global population (Healton et al., 2006; Rupprecht et al., 2015; Dare et al., 2015; Roberts and Rosenheck, 2020).

In 2021, approximately 11% of individuals who were 12 years old or older (approximately 4.3 million) used a tobacco or nicotine product in the US in the past month (SAMHSA, 2021). Tobacco use alone leads to the development of a multitude of health issues and is one of the leading causes of death for US citizens and costs nearly $600 billion annually (CDCTobaccoFree, 2021). Additionally, tobacco use is associated with increased incidence of other substance use issues (Schulte, et al., 2014; Smith et al., 2014), as well as greater relapse rates in those with other substance use disorders (SUDs; Weinberger et al., 2017). Approximately 35% of smokers meet criteria for a psychiatric condition (CDC, 2020). However, it should be noted that for individuals expressing multiple mental health disorders, smoking rates can be as high as 60%+ (Prochaska et al., 2017).

Obesity, as defined as a BMI of 30 kg/m^2^ or above, in the United States has a reported prevalence of nearly 42% in the adult population with a reported annual medical cost of over $147 billion (CDC, 2022). Obesity increases the risk of numerous diseases (e.g., heart disease, diabetes) and is linked to nearly 2.8 million deaths worldwide annually (WHO, 2021). In regards to mental health, for persons with obesity, mood and anxiety disorders appear most prevalent, with an approximate 25% increase in risk of either when compared to individuals at lower weight ranges (Simon et al., 2006; Mather et al., 2009; Luppino et al., 2010). These disorders also appeared to be more prevalent in women with obesity compared to men with obesity (Scott et al., 2008).

Some evidence suggests that co-occurrence of obesity and TUD can put individuals at an even greater risk for disease and psychiatric conditions than does either condition occurring alone. Indeed, a recent report published by Roberts and Rosencheck (2020) based on national data from Veterans treated in the Veterans Health Administration (VHA) showed that individuals who were obese and also diagnosed with a TUD had higher rates of comorbid health and psychiatric conditions (e.g. bipolar and personality disorder) than either TUD or obesity alone. However, a surprising finding in the same study, revealed that those individuals who were characterized as obese had the lowest risk of a comorbid SUD. These results suggest the possibility that people with obesity, even those with a comorbid TUD, may have an overall reduced risk for SUD diagnosis when compared to individuals diagnosed with a TUD alone. Similarly, a study conducted by Pickering et al. (2011) found that both men and women who were either categorized as overweight or obese were at a decreased risk for being diagnosed with alcohol use disorder (AUD) and some other SUDs. Though the exact mechanism for this reduced risk of substance use issues in individuals who are obese remains somewhat unclear, it is speculated that the reward pathways that mediate the reinforcing properties of drugs of abuse are activated in a similar and likely competitive manner by highly rewarding foods; often referred to as the food-drug competition hypothesis (Kleiner et al., 2004; Warren and Gold, 2007; Nolan, 2013; Sansone and Sansone, 2013; Volkow and Baler, 2015; Michaud et al., 2017). Additional support for this phenomenon comes from the food-drug substitution hypothesis where individuals may substitute one highly rewarding substance, such as food, with another, such as drugs of abuse (Ivezaj et al., 2012; Kim et al., 2021). This appears to be particularly common in post-operative bariatric patients (Azam et al., 2018). These theories have been used to support the proposal of food addiction as a distinct clinical diagnosis, but further research is needed (Shaffer et al., 2004; Barry et al., 2009; Avena et al., 2012; Gearhardt et al., 2018; Hoover et al., 2022; Vasiliu, 2022). These theories, however, do not support the co-expression of both conditions. However, this may be explained by dysregulated reward sensitivity which is particularly common in more extreme BMI ranges (Reynolds, 2006; Stanger et al., 2013; Rupprecht et al., 2015). Additionally, the distinct neurobiological pathways of both TUD and obesity, which ultimately lead to activation of the dopaminergic reward system, may also contribute to their co-occurrence (Benowitz, 2009; Berthoud, 2002). The co-expression of obesity with TUD is further complicated by the appetite suppressing effects of nicotine (Audrain-McGovern and Benowitz, 2011); however people who smoke, including those with obesity, often report that weight management is a motive for their continued tobacco use (White et al., 2007; White, 2012).

The current study aims to extend the findings originally reported in Roberts and Rosenheck (2020) by further characterizing multimorbid expression of TUD and obesity using the nationally representative NESARC-III dataset which uses the gold-standard AUDADIS (Grant et al., 2014). We hypothesized that people with TUD alone will be more likely to meet criteria for multimorbid SUD compared to those with TUD whose BMI falls in the obese range. Additionally, based on previous findings (Pickering et al., 2011; Roberts and Rosenheck, 2020), we expected that people with TUD and obesity would show higher rates of other psychiatric disorders compared to those with either disorder alone. The prior study investigating this specific comorbidity relied on health records from the VHA, which have limitations due to non-randomized sampling and reliance on unstandardized diagnoses (Vassar and Holzmann, 2013). It is important to address these limitations using alternative and representative data sources to confirm the veracity of these surprising results. Additionally, the previous study did not include a healthy comparison group (i.e., those without either obesity or tobacco use), and such a comparison group helps to better contextualize the risk associated with each condition and their multimorbid expression.

## 2 Materials and methods

### 2.1 Data source

Data for this study was collected from the NESARC-III, which is a national survey conducted by the National Institute on Alcohol Abuse and Alcoholism (NIAAA) (Grant et al., 2014). The sample (*n* = 36,309) included noninstitutionalized civilians who were 18 years or older from the United States. Participants who gave informed consent completed an in-person computer assisted interview (Alcohol Use Disorder and Associated Disabilities Interview Schedule-5; AUDADIS-5). The AUDADIS-5 is a valid measure of SUDs and TUDs (Hasin et al., 2015). Data weights were applied to adjust for oversampling and nonresponse. Additional methodological details on the NESARC-III are provided elsewhere (Grant et al., 2014).

### 2.2 Covariates/demographics

Demographic variables were selected because of their probable association with obesity and TUD and included sex, race/ethnicity, age, income, nativity, marital status, census region, urban/rural residence, and education. These demographic covariates were included in all models. All demographic variables were treated as categorical variables except income. Income was treated as a continuous category ranging from 1 (>$4999) to 17 (<$100,000). Please see Table 1 for a detailed breakdown of all demographic characteristics.

**Table 1:** Weighted demographic variables.

	1. Comparison Group	2. TUD	3. Obese	4. TUD + Obese
**Sex**	%	%	%	%
Male	46.76	57.05	46.12	53.86
Female	53.24	42.95	53.88	46.14
**Race/Ethnicity**	%	%	%	%
White, non-Hispanic	65.11	75.24	62.45	70.4
Black, non-Hispanic	9.84	10.66	16.06	14.63
American Indian/Alaska Native, non-Hispanic	1.11	2.220	1.98	2.52
Asian/Native Hawaiian/Other Pacific Islander, non-Hispanic	8.31	3.64	2.110	2.17
Hispanic, any race	15.61	8.23	17.4	10.27
**Age**	%	%	%	%
< 24	12.93	16.57	7.85	8.78
25 to 34	16.59	22.68	14.45	22.42
35 to 44	15.87	16.94	18.54	23.75
45 to 59	24.73	29.5	30.42	33.76
60+	27.87	14.3	28.74	11.3
**Income**	M (SE)	M (SE)	M (SE)	M (SE)
	7.85 (0.074)	6.52 (0.089)	7.73 (0.080)	6.71 (0.148)
**Nativity**	%	%	%	%
United States	79.55	92.93	86.8	94.58
Non-United States	20.45	7.07	13.19	5.42
**Marital Status**	%	%	%	%
Married	53.62	34.8	57.1	43.9
Living with someone as if married	5.69	10.58	5.36	11.86
Widowed	6.54	4.2	5.66	2.77
Divorced	8.82	16.68	11.34	15.04
Separated	2.35	4.63	2.78	4.52
Never married	22.97	29.11	17.76	21.91
**Census Region**	%	%	%	%
Northeast	19.33	16.38	16.7	17.77
Midwest	19.62	24.1	23.430	24.800
South	35.270	39.910	38.91	40.03
West	25.78	19.6	20.95	17.4
**Urban or Rural Residence**	%	%	%	%
Urban	81.64	73.63	76.74	72.39
Rural	18.36	26.37	23.26	27.61
**Education**	%	%	%	%
Less than high school	10.77	18.19	13.55	18.06
High school	22.25	33.27	27.22	34.44
Some college	31.03	35.54	35.41	36.54
Bachelor's degree	22.55	9.48	15.57	8.03
Graduate degree	13.39	3.58	8.25	2.93

### 2.3 Classification strategy

Participants were stratified into the following four groups based on their BMI and TUD status:

*Comparison*: Participants who did not meet the past-year criteria for a TUD and had a BMI below 30 kg/m^2^ were categorized into the comparison group.
*TUD:* Those individuals classified as TUD were those who met the DSM-5 criteria for a TUD in the last year using the AUDADIS-5 and also had a BMI < 30 kg/m^2^.
*Obese:* Participants’ self-reported height and weight were used to calculate BMI. A person with obesity was defined as having a BMI of greater than or equal to 30 (calculated as weight in kilograms divided by the square of an individual's height in meters) which is equivalent to class 1 obesity or greater (CDC, 2022).
*TUD + Obese:* Participants who met the past-year criteria for TUD (as described above) and had a BMI greater than or equal to 30 kg/m^2^ were classified as an individual with obesity and a TUD.

### 2.4 Substance use

DSM-5 SUDs were evaluated using the AUDADIS. All SUD diagnoses were based on symptoms occurring in the past 12 months. Results included AUD, cannabis use disorder (CUD), other SUDs (i.e., inhalant/solvent, club drug, heroin, hallucinogen, stimulant, or sedative). We also calculated a SUD count outcome that indicated how many separate SUDs for which the individual met criteria.

### 2.5 Mental health

DSM-5 mental health disorders were evaluated using the AUDADIS. Diagnosis for each disorder was based on symptoms occurring in the past 12 months. Results included diagnoses for mood disorders (i.e., major depressive, dysthymia, bipolar 1), anxiety disorders (i.e., agoraphobia, panic disorder, social phobia, specific phobia, generalized anxiety, and posttraumatic stress) and personality disorders (i.e., schizotypal, borderline, antisocial personality). We also included a count variable that indicated the number of diagnoses for which the individual met criteria.

### 2.6 Missing data

In cases where participants are missing demographic variables (e.g., age, race) NESARC-III adds logically assigned values (detailed method of logical assignment is reported in Grant et al., 2014). Missing data rates were low (*n*=552, 1.5% of sample with some missing data). Height and weight were the most common missing variables. We used a complete case analysis approach, such that participants with any missing data were excluded from further analysis.

### 2.7 Statistical analysis

Models were generated using the “survey” package in R (version 4.1.3). All covariates discussed above were included in all analyses (i.e., sex, race/ethnicity, age, income, nativity, marital status, census region, urban/rural residence, and education). The primary analyses examined the differences between individuals who met criteria for obesity, TUD, or both (obesity +TUD) to a comparison group on a variety of SUDs and Mental Health Disorders (described above). All analyses incorporated the stratification, clustering, and unequal weighting of the sampling design. Binary logistic regression was used to analyze binary nominal outcome variables (i.e., psychiatric conditions, any SUD). All count based outcomes were analyzed using quasipoisson regression. We report adjusted odds ratios (aOR; logistic models) and adjusted rate ratios (aRR; quasipoisson models) and 95% confidence intervals. *A priori* pairwise contrasts were conducted to probe the relative differences in risk/rate between groups. The significance threshold for these contrasts was set at *p*<.05. We also conducted a supplemental analysis to examine these associations among people with different degrees of obesity (i.e., underweight, healthy weight, overweight, and Class 1 & 2). While interpreting these models is limited by the proliferation of groups limiting cell sizes, we include them to provide some insight into how obesity severity may affect the described associations. These models are presented in a supplemental table.

## 3 Results

### 3.1 Sample characteristics

All demographic information is presented in Table 1. The sample included 36,309 individuals. 2135 (5.88%) individuals were diagnosed with a TUD and also reported BMI in the obese range (30+ kg/m^2^; TUD + obesity). 8845 (24.36%) individuals met criteria for obesity but did not meet TUD criteria (obese alone). 5170 (14.24%) individuals met criteria for a diagnosis of TUD with a BMI below 30kg/m^2^ (TUD alone). 20,159 (55.52%) individuals had no TUD and a BMI <30kg/m^2^ (Comparison). Respondents were primarily White (66.22%), with men and women representing roughly equal proportions (48.48% and 51.51% respectively).

### 3.2 Substance use disorders

Table 2 presents results comparing the individuals described above (Comparison, TUD, Obese, and TUD + Obesity) on past year SUDs. Relative to comparison individuals, those individuals with a TUD alone (aOR= 3.53) were significantly more likely to meet criteria for any SUD followed by individuals with TUD + obesity (aOR= 2.55). Individuals with obesity alone did not differ from comparison adults on SUDs. Similarly, when compared to comparison individuals, individuals with TUD alone (aRR = 2.60) had the greatest number of diagnosed SUDs followed by individuals with TUD + obesity (aRR = 2.19). Again, individuals with obesity alone and comparison individuals did not differ in the number of diagnosed SUDs.

**Table 2:** SUD diagnosis rates.

	1. Comparison	2. TUD			3. Obese			4. TUD + Obese			
	% (SE)	% (SE)	aOR	95% CI	% (SE)	aOR	95% CI	% (SE)	aOR	95% CI	Contrasts
**Any Substance Use Disorder**	11.72 (0.35)	35.18 (1.08)	3.53	3.19; 3.91	9.38 (0.36)	0.89	0.80; 0.99	26.75 (1.39)	2.55	2.19; 2.96	2 > 4 > 1, 3
**Alcohol Use Disorder**	10.94 (0.33)	30.99 (1.04)	3.17	2.86; 3.51	8.69 (0.35)	0.89	0.80; 0.99	23.39 (1.3)	2.32	1.99; 2.70	2 > 4 > 1, 3
**Cannabis Use Disorder**	1.24 (0.09)	8.79 (0.53)	6.55	5.34; 8.03	1.01 (0.11)	1.02	0.78; 1.34	6.55 (0.71)	5.69	4.30; 7.53	2, 4 > 3, 1
**Other Drug**	0.44 (0.06)	3.03 (0.29)	0.48	0.04; 6.29	0.27 (0.05)	1.41	0.47; 4.21	2.29 (0.459)	2.56	0.43; 15.03	NSC
	M (SE)	M (SE)	aRR	95% CI	M (SE)	aRR	95% CI	M (SE)	aRR	95% CI	Contrast
**Substance Use Disorders (#)**	0.12 (0.004)	0.39 (0.01)	2.60	2.43; 2.78	0.09 (0.004)	0.9	0.83; 0.99	0.30 (0.02)	2.19	1.96; 2.44	2 > 4 > 1, 3

Note: TUD = tobacco use disorder, aOR = adjusted odds ratio, aRR = adjusted rate ratios, NSC = no significant contrasts, SE = standard error, CI = confidence intervals, *M* = mean. All groups represented in the contrasts column met the significance threshold which was set at *p*<.05.

In examining individual substance categories, relative to comparison adults, individuals with TUD alone (aOR = 3.17) and individuals with TUD + obesity (aOR = 2.32) were significantly more likely to be diagnosed with AUD. Individuals with obesity alone did not differ from comparison individuals. Individuals with TUD alone (aOR = 6.55) and individuals with TUD + obesity (aOR = 5.69) were also significantly more likely to be diagnosed with CUD than comparison or individuals with obesity alone. There was no evidence of significant group differences in rates of other SUDs.

### 3.3 Comparison of mental health

Rate of diagnosis for past year mental health disorders as defined by DSM-5 criteria are described in Table 3. For the majority of disorders examined (with the exception of dysthymia, agoraphobia, specific phobia and antisocial personality disorder) there was no significant difference in prevalence rates between individuals with TUD + obesity and individuals with TUD alone. Additionally, individuals with obesity alone generally had a significantly lower likelihood of diagnosis than either individuals with TUD alone or individuals with TUD + obesity (with the exception of dysthymia); although they had higher rates of diagnosis than comparison adults.

**Table 3:** Psychiatric disorder diagnosis rate.

	1. Comparison	2. TUD			3. Obese			4. TUD + Obese			
MOOD DISORDERS	% (SE)	% (SE)	aOR	95% CI	% (SE)	aOR	95% CI	% (SE)	aOR	95% CI	Contrasts
Major Depressive Disorder	7.91 (0.25)	16.74 (0.69)	2.2	1.94; 2.49	10.53 (0.41)	1.4	1.26; 1.57	17.84 (1.15)	2.48	2.07; 2.96	4, 2 > 3 > 1
Dysthymia	2.15 (0.12)	5.11 (0.40)	2	1.62; 2.45	3.36 (0.21)	1.61	1.37; 1.91	6.17 (0.60)	2.63	2.10; 3.29	4 > 2, 3 >1
Bipolar 1 Disorder	0.70 (0.05)	4.02 (0.38)	4.81	3.74; 6.19	1.20 (0.15)	1.72	1.29; 2.29	4.88 (0.65)	6.18	4.35; 8.78	4, 2 > 3 > 1
**ANXIETY DISORDERS**	% (SE)	% (SE)	aOR	95% CI	% (SE)	aOR	95% CI	% (SE)	aOR	95% CI	Contrasts
Agoraphobia	0.90 (0.07)	3.18 (0.33)	3.23	2.37; 4.41	1.53 (0.17)	1.72	1.32; 2.25	3.22 (0.69)	3.36	2.11; 5.35	4, 2 > 3 > 1
Panic Disorder	1.82 (0.12)	6.65 (0.38)	3.37	2.85; 3.98	2.83 (0.24)	1.54	1.25; 1.90	7.13 (0.77)	3.64	2.81; 4.71	4, 2 > 3 > 1
Social Phobia	2.06 (0.14)	4.95 (0.32)	1.96	1.65; 2.34	2.86 (0.22)	1.34	1.12; 1.60	5.21 (0.58)	2.15	1.63; 2.84	4, 2 > 3 > 1
Specific Phobia	4.61 (0.21)	8.47 (0.43)	1.8	1.55; 2.09	6.16 (0.33)	1.33	1.12; 1.57	7.67 (0.68)	1.59	1.29; 1.95	2 > 3, 4 > 1
Generalized Anxiety Disorder	3.76 (0.17)	9.10 (0.43)	2.52	2.21; 2.88	5.47 (0.27)	1.53	1.33; 1.75	10.16 (0.96)	2.92	2.32; 3.67	4, 2 > 3 > 1
Posttraumatic Stress Disorder	2.96 (0.15)	8.82 (0.58)	2.71	2.25; 3.26	4.74 (0.26)	1.55	1.32; 1.82	10.54 (0.80)	3.3	2.72; 4.01	4, 2 > 3 > 1
**PERSONALITY DISORDERS**	% (SE)	% (SE)	aOR	95% CI	% (SE)	aOR	95% CI	% (SE)	aOR	95% CI	Contrasts
Schizotypal Personality Disorder	2.80 (0.14)	9.32 (0.52)	2.59	2.25; 2.98	3.93 (0.223)	1.34	1.15; 1.56	9.40 (0.86)	2.73	2.14; 3.48	4, 2 > 3 > 1
Borderline Personality Disorder	6.19 (0.22)	21.99 (0.84)	3.23	2.90; 3.60	9.01 (0.44)	1.43	1.27; 1.62	23.21 (1.17)	3.6	3.06; 4.24	4, 2 > 3 > 1
Antisocial Personality Disorder	1.37 (0.12)	6.95 (0.48)	3.51	2.90; 4.25	1.90 (0.15)	1.28	1.02; 1.61	6.47 (0.78)	3.37	2.41; 4.72	4, 2 > 3 > 1
	% (SE)	% (SE)	aRR	95% CI	% (SE)	aRR	95% CI	% (SE)	aRR	95% CI	Contrasts
**Mental Health Disorders (#)**	0.37 (0.01)	1.05 (0.03)	2.38	2.22; 2.55	0.53 (0.02)	1.4	1.31; 1.51	1.12 (0.05)	2.59	2.33; 2.87	4, 2 > 3 > 1

Note: TUD = tobacco use disorder, aOR = adjusted odds ratio, aRR = adjusted rate ratios, NSC = no significant contrasts, SE = standard error, CI = confidence intervals, *M* = mean. All groups represented in the contrasts column met the significance threshold which was set at *p*<.05.

For dysthymia, relative to comparison individuals, individuals with TUD + obesity (aOR = 2.33), individuals with TUD alone (aOR = 1.83), and individuals with obesity alone (aOR = 1.54) all had a higher likelihood of diagnosis. Additionally individuals with TUD + obesity had a greater likelihood of diagnosis than individuals with obesity alone, but not individuals with TUD alone. For agoraphobia, relative to comparison individuals, individuals with TUD alone (aOR = 2.87), individuals with TUD + obesity (aOR = 2.8), and individuals with obesity alone (aOR = 1.65) all had a higher likelihood of diagnosis. Additionally individuals with TUD alone had a greater likelihood of diagnosis than individuals with obesity alone, but not individuals with TUD + obesity. For specific phobia, relative to comparison individuals, individuals with TUD alone (aOR = 1.78), individuals with TUD + obesity (aOR = 1.57), and individuals with obesity alone (aOR = 1.33) all had a higher likelihood of diagnosis. Additionally individuals with TUD alone had a greater likelihood of diagnosis than individuals with obesity alone, but not individuals with TUD + obesity. For antisocial personality disorder, individuals with TUD alone (aOR = 3.17) and individuals with TUD + obesity (aOR = 2.95) had a higher likelihood of diagnosis than both individuals with obesity alone (aOR=1.26) and comparison individuals. Finally, individuals with TUD + obesity (aRR = 2.38) and individuals with TUD alone (aRR = 2.24) experienced the highest rates of mental health disorders, followed by individuals with obesity alone (aRR = 1.4), and then comparison adults

## 4 Discussion

The current study aimed to document substance use and psychiatric comorbidities among people with TUD, obesity, and the combination of these health conditions. Consistent with our hypotheses, we found that people with obesity, including those with TUD, had lower rates of comorbid SUD diagnosis than those with TUD alone. These findings are consistent with other studies that investigated the relationship between obesity and the development of SUDs (Healton et al., 2006; Dare et al., 2015; Gearhardt et al., 2012, 2018). For instance, Pickering et al. (2011) found in Waves I and II of the NESARC that a higher BMI was inversely related with AUD risk. Evidence also supports the reduction of comorbid SUDs even in the presence of TUD. In fact, Roberts and Rosenheck (2020) found that veterans treated in VHA with class 3 obesity (those who were at a BMI ≥ 40kg/m^2^) with or without a TUD were less likely to be diagnosed with another SUD compared to those of healthy weight who used tobacco. Both of these reports, along with the current findings, support the notion that obesity reduces the risk of the development of substance use problems, even among people who possess other risk factors (e.g., tobacco use). Finally, participants with TUD but no obesity had the comparatively highest rate of comorbid SUD diagnosis, consistent with previous studies showing that TUD is a major risk factor for SUD's (Mckee et al., 2007; Smith et al., 2014; John et al., 2018).

The precise mechanisms behind the lower rates of substance use disorders (SUD) in individuals with obesity remain somewhat elusive. It is believed that both conditions share similar neurobiological circuits that contribute to their development (Volkow and Wise, 2005; Volkow et al., 2011). This commonality may create a competitive relationship between rewarding food and substances of abuse, potentially reducing the need for one when the other is present (Kleiner et al., 2004; Warren and Gold, 2007; Nolan, 2013; Sansone and Sansone, 2013; Michaud et al., 2017). This phenomenon is often referred to as the food-drug competition hypothesis (Cummings et al., 2015; Gearhardt et al., 2012, 2018). The competition appears to be centered around dopaminergic reward pathways involved in reinforcement, decision-making, craving, and delay discounting (Volkow and Baler, 2015; Poisson et al., 2021). This food-drug competition hypothesis may explain why, in the current study, individuals with obesity had the lowest rate of comorbid SUD diagnoses.

However, among those with both conditions, the rate of comorbid SUDs was lower than in those with a tobacco use disorder (TUD) alone but still significantly higher than in those with obesity alone. Obesity might contribute to a reduction in comorbid SUDs through food-drug competition, but it is possible that these individuals have a dysregulated reward pathway (Reynolds, 2006; Stanger et al., 2013; Rupprecht et al., 2015), where the competition between food and drugs exists but is less prominent. Further research is clearly needed to disentangle the association between obesity and SUD risk.

We found limited support for our hypothesis that people with TUD and obesity would show higher rates of psychiatric comorbidity. Generally, these individuals did have the highest rates of psychiatric disorders when compared to others in our analyses, but the difference between them and those expressing only a TUD was mostly non-significant (with the exception of dysthymia). Additionally, the reduced risk conferred by obesity regarding the development of SUDs does not appear to extend beyond SUDs into other categories of behavioral health. Indeed, we found that people who meet obesity criteria are at a higher risk for most psychiatric disorders compared to those of healthy weight, consistent with prior research (for review see Blüher, 2019; Skou et al., 2022). Obesity and TUD individually are associated with an increased prevalence of mental health conditions (Simon et al., 2006; Smith et al., 2014) with TUD more often co-occurring with multiple mental health disorders (Prochaska et al., 2017). However, comorbid expression of these conditions, or any comorbidity, appears to make mental health issues worse (Roberts and Rosenheck, 2020; Skou et al., 2022). Perhaps the use of BMI as a dichotomous classification of obese or not in the current study may have limited findings, as the relationship between BMI, TUD, and other conditions may be more nuanced (Rupprecht et al., 2015). For instance, a U-shaped relationship between BMI and TUD has been observed, where those with either low or high BMI had higher rates of TUD, while those with moderate BMI had lower rates (Chatkin et al., 2010). Therefore, comparing only healthy weight (BMI ≤ 29kg/m^2^) and people with obesity (BMI ≥ 30kg/m^2^) may oversimplify the relationship. Furthermore, the amount of smoking may also play a role in the relationship between TUD and mental health, as those who consume the most cigarettes often have more severe mental health conditions (Prochaska et al., 2017; Smith et al., 2014). Further research is needed to address these questions, however, it is clear that clinical guidelines should continue to focus on serving individuals who either express or are at risk for multimorbidities because it commonly leads to worse outcomes for the patients over the lifespan.

This study builds on Roberts and Rosenheck (2020) by further examining the relationship between multimorbid obesity, TUD, and the development of SUD and other psychiatric conditions. The previous study had limitations due to its reliance on VHA health records, potentially reducing generalizability (Vassar and Holzmann, 2013). In contrast, the current study used the NESARC-III which relies on a systematic, representative sampling strategy and used the gold-standard AUDADIS survey instrument. Moreover, it included a comparison/control group and defined obesity as class 1 or greater (BMI > 30kg/m^2^), possibly contributing to the differences in findings. However, the consistent results related to SUD across methodologies demonstrate the robustness of these findings (Roberts and Rosenheck, 2020).

This study offers valuable insights into the health risks of obesity and TUD but has some limitations. The cross-sectional data prevents direct exploration of underlying mechanisms, requiring longitudinal or human laboratory studies for clarification (Buis and Van Roosmalen, 2021). Additionally, the use of BMI as the primary indicator of dysregulated eating behaviors may limit understanding, as it does not encompass all potential variables affecting body fat (Rothman, 2008; Nuttall, 2015). Future studies could benefit from more comprehensive measures. However, BMI is practical for large-scale population studies (Gutin, 2018). We elected to operationalize obesity categorically in the current study to support direct clinical translation of our findings. There may, however, be advantages to treating BMI continuously in terms of uncovering more nuanced associations between obesity and behavioral health conditions (Lohse et al., 2017). In regards to the data, NESARC-III has been criticized as potentially overestimating the rate of SUD in the population (Caetano, 2015), which may result in overestimation of rates of multimorbidity in the cohorts we examined here. Lastly, NESARC-III data was collected in 2012–2013, and population-level shifts in outcomes may have occurred since then.

## 5 Conclusions

This study demonstrates that obesity is associated with a decreased risk of developing SUD, even in individuals with other risk factors, but is still associated with other health-related disorders. These findings have implications for targeted prevention efforts and intervention development for treating obesity, SUD's, or both. Clinicians may use this information to tailor treatment plans, considering the potential development of additional SUDs and psychiatric disorders in those with either or both conditions. As multimorbidities contribute to poorer health outcomes, especially in low SES communities, understanding risk factors and providing comprehensive treatment is crucial, particularly considering that obesity rates appear to be on the rise (Skou et al., 2022; Aggarwal et al., 2023). These results also provide further evidence for the food-drug competition hypothesis which lends additional support for a unique diagnostic category for food addiction. However, future studies employing pre-clinical, clinical, or longitudinal methods are needed to investigate the mechanisms of these associations. Further characterization of the relationships, risk factors, and temporal dynamics between obesity and SUD could help elucidate the complex interaction between these conditions and inform clinical practice.

## Funding source

This work was supported by NIAAA grants K01AA025670 (TLV), R03AA028361 (TLV), K23AA026890 (WR), U54AA027989 (SAM), and a VA VISN1 CDA and PASA (2022RFA6a; MRP).

## Declaration of Competing Interest

Given her role as Associate Editor, Sherry McKee, PhD had no involvement in the peer-review of this article and has no access to information regarding its peer-review. Full responsibility for the editorial process for this article was provided by Teri Franklin, PhD.

## Appendix Supplementary materials

Image, application 1
