# Supplementary material for: Examining the relationship of concurrent obesity and tobacco use disorder on the development of substance use disorders and psychiatric conditions: Findings from the NESARC-III
Source: Drug Alcohol Depend Rep. 2023 Apr 23;7:100162. doi: 10.1016/j.dadr.2023.100162 (PMC10163607; doi:10.1016/j.dadr.2023.100162)
Supplement: Supplementary file 1 [file mmc1.docx]

Supplemental Table 1: Sample distribution with expanded obesity categories

| Sample Distribution (%) |  |  |
| --- | --- | --- |
|  | No-TUD | TUD |
| 1. BMI < 18.5 | 1.17 | 0.45 |
| 2. BMI 18.5 - 25 | 26.79 | 7.22 |
| 3. BMI 25 - 30 | 27.56 | 6.56 |
| 4. BMI 30 -35 | 14.67 | 3.59 |
| 5. BMI 35 + | 9.68 | 2.27 |

Note: BMI (calculated as kg/m2) ranges correspond to the accepted BMI classification system used by the CDC (CDC, 2020).

Supplemental Table 2. Weighted demographic variables with expanded obesity categories

|  | No TUD |  |  |  |  | TUD |  |  |  |  |
| --- | --- | --- | --- | --- | --- | --- | --- | --- | --- | --- |
| **SEX** | 1. BMI < 18.5 | 2. BMI 18.5 - 25 | 3. BMI 25 - 30 | 4. BMI 30 -35 | 5. BMI 35 + | 6. BMI < 18.5 | 7. BMI 18.5 - 25 | 8. BMI 25 - 30 | 9. BMI 30 -35 | 10. BMI 35 + |
| (Male/Female) % | 25.63 / 74.37 | 38.67 / 61.34 | 55.52 / 44.48 | 50.12 / 49.87 | 40.06 / 59.94 | 29.72 / 70.27 | 53.04 / 46.95 | 63.33 / 36.67 | 56.67 / 43.32 | 49.4 / 50.58 |
| Race |  |  |  |  |  |  |  |  |  |  |
| White, non-Hispanic | 58.47 | 65.91 | 64.62 | 63.04 | 61.55 | 79.96 | 76.01 | 74.06 | 71 | 69.44 |
| Black, non-Hispanic | 6.73 | 8.42 | 11.36 | 14.69 | 18.12 | 8.01 | 9.94 | 11.62 | 14.72 | 4.49 |
| American Indian/Alaska Native, non-Hispanic | 0.13 | 1.12 | 1.13 | 1.56 | 2.6 | 0.68 | 2.19 | 2.34 | 2.35 | 2.77 |
| Asian/Native Hawaiian/Other Pacific Islander, non-Hispanic | 20.45 | 10.72 | 5.41 | 2.54 | 1.45 | 2.95 | 4.07 | 3.21 | 2.56 | 1.54 |
| Hispanic, any race | 13.91 | 13.78 | 17.45 | 18.15 | 16.25 | 8.38 | 7.76 | 8.73 | 9.34 | 11.73 |
| **Age** |  |  |  |  |  |  |  |  |  |  |
| < 24 | 30.61 | 20.48 | 8.86 | 8.69 | 6.56 | 20.55 | 21.34 | 11.04 | 8.81 | 8.73 |
| 25 to 34 | 17.95 | 18.35 | 14.81 | 13.87 | 15.31 | 17.55 | 24.19 | 21.38 | 21.42 | 23.99 |
| 35 to 44 | 14.39 | 14.86 | 16.91 | 17.35 | 20.34 | 12.95 | 15.02 | 19.32 | 22.12 | 26.32 |
| 45 to 59 | 13.86 | 21.37 | 28.46 | 29.19 | 32.27 | 26.07 | 25.82 | 33.79 | 34.94 | 31.88 |
| 60+ | 23.18 | 24.91 | 30.94 | 30.88 | 25.49 | 22.85 | 13.61 | 14.45 | 12.71 | 9.05 |
| **Income** |  |  |  |  |  |  |  |  |  |  |
|  | 5.24 | 7.35 | 8.44 | 7.87 | 7.51 | 4.66 | 6.01 | 7.21 | 7.02 | 6.22 |
| **Nativity** |  |  |  |  |  |  |  |  |  |  |
| United States | 72.47 | 79.35 | 80.04 | 84.61 | 90.12 | 90.72 | 93.29 | 92.68 | 93.37 | 96.47 |
| Non-United States | 27.52 | 20.64 | 19.95 | 15.38 | 9.87 | 9.27 | 6.71 | 7.31 | 6.62 | 3.52 |
| **Marital Status** |  |  |  |  |  |  |  |  |  |  |
| Married | 39.95 | 49.02 | 58.67 | 58.59 | 54.83 | 28.25 | 30.79 | 39.66 | 45.83 | 40.83 |
| Living with someone as if married | 5.89 | 5.82 | 5.56 | 5.36 | 5.37 | 3.11 | 11.27 | 10.32 | 11.71 | 12.09 |
| Widowed | 9.02 | 6.42 | 6.53 | 5.52 | 5.81 | 10.58 | 4.2 | 3.75 | 2.34 | 3.45 |
| Divorced | 7.55 | 8.48 | 9.19 | 11.02 | 11.8 | 16.01 | 16.32 | 17.11 | 15.02 | 15.06 |
| Separated | 2.26 | 2.19 | 2.51 | 2.66 | 2.95 | 8.63 | 4.58 | 4.39 | 4.63 | 4.34 |
| Never married | 35.31 | 28.05 | 17.51 | 16.84 | 19.15 | 33.4 | 32.81 | 24.75 | 20.45 | 24.21 |
| **Census Region** |  |  |  |  |  |  |  |  |  |  |
| Northeast | 17.44 | 20.36 | 18.41 | 16.05 | 17.68 | 13.44 | 17.38 | 15.47 | 18.61 | 16.43 |
| Midwest | 14.76 | 19.75 | 20.25 | 22.56 | 24.74 | 20.56 | 22.26 | 26.37 | 26.11 | 22.72 |
| South | 35.96 | 33.31 | 37.15 | 38.96 | 38.82 | 44.81 | 39.83 | 39.65 | 37.43 | 44.11 |
| West | 31.81 | 27.15 | 24.18 | 22.41 | 18.73 | 21.18 | 20.51 | 18.49 | 17.84 | 16.71 |
| **Urban or Rural Residence** |  |  |  |  |  |  |  |  |  |  |
| Urban | 85.89 | 83.64 | 79.51 | 77.16 | 76.09 | 74.08 | 73.95 | 73.23 | 73.82 | 70.11 |
| Rural | 14.11 | 16.35 | 20.48 | 22.84 | 23.91 | 25.91 | 26.04 | 26.76 | 26.17 | 29.88 |
| **Education** |  |  |  |  |  |  |  |  |  |  |
| Less than high school | 11.01 | 9.66 | 11.84 | 13.07 | 14.25 | 24.08 | 18.87 | 17.02 | 16.95 | 19.79 |
| High school | 22.55 | 20.85 | 23.59 | 27.16 | 27.31 | 36.36 | 33.18 | 33.14 | 33.73 | 35.54 |
| Some college | 30.41 | 31.07 | 31.01 | 35.17 | 35.77 | 36.98 | 34.74 | 36.33 | 36.31 | 36.91 |
| Bachelor's degree | 26.86 | 24.42 | 20.54 | 16.11 | 14.74 | 2.25 | 9.62 | 9.78 | 9.95 | 4.98 |
| Graduate degree | 9.16 | 13.97 | 13.01 | 8.47 | 7.92 | 0.31 | 3.56 | 3.71 | 3.04 | 2.75 |

Note: BMI (calculated as kg/m2) ranges correspond to the accepted BMI classification system used by the CDC (CDC, 2020).

Supplemental Table 3. Substance Use Disorders with expanded obesity categories:

|  |  | **No TUD** |  |  |  | **TUD** |  |  |
| --- | --- | --- | --- | --- | --- | --- | --- | --- |
| **Any SUD** | %(SE) | aOR | 95% CI |  | %(SE) | aOR | 95% CI | Contrasts |
| 1. BMI < 18.5 | 10.84 (1.83) | 0.82 | 0.55; 1.21 | 6. BMI < 18.5 | 29.48 (4.88) | 3.41 | 2.01; 5.77 | 6-10 > 1-5 |
| 2. BMI 18.5 - 25 | 12.85 (0.46) | REF | REF | 7. BMI 18.5 - 25 | 37.94 (1.24) | 3.73 | 3.31; 4.21 | 7 > 9, 10 |
| 3. BMI 25 - 30 | 10.65 (0.40) | 0.98 | 0.88; 1.08 | 8. BMI 25 - 30 | 32.54 (1.35) | 3.21 | 2.78; 3.69 | 8 >10 |
| 4. BMI 30 -35 | 9.58 (0.50) | 0.9 | 0.78, 1.03 | 9. BMI 30 -35 | 28.22 (1.70) | 2.72 | 2.27; 3.25 |  |
| 5. BMI 35 + | 9.09 (0.48) | 0.84 | 0.74; 0.95 | 10. BMI 35 + | 24.42 (1.95) | 2.19 | 1.75; 2.75 |  |
| **AUD** |  |  |  |  |  |  |  |  |
| 1. BMI < 18.5 | 8.66 (1.67) | 0.7 | 0.45; 1.07 | 6. BMI < 18.5 | 25.68 (4.59) | 3.08 | 1.8; 5.25 | 6-10 > 1-5 |
| 2. BMI 18.5 - 25 | 11.96 (0.43) | REF | REF | 7. BMI 18.5 - 25 | 32.72 (1.24) | 3.21 | 2.85; 3.63 | 5 > 2 |
| 3. BMI 25 - 30 | 10.04 (0.41) | 0.98 | 0.88; 1.10 | 8. BMI 25 - 30 | 29.45 (1.30) | 3.01 | 2.61; 3.47 | 10 > 7, 8 |
| 4. BMI 30 -35 | 9.08 (0.51) | 0.92 | 0.79; 1.06 | 9. BMI 30 -35 | 24.87 (1.66) | 2.48 | 2.05; 2.99 |  |
| 5. BMI 35 + | 8.10 (0.45) | 0.8 | 0.71; 0.91 | 10. BMI 35 + | 21.07 (1.70) | 1.99 | 1.59; 2.49 |  |
| **CUD** |  |  |  |  |  |  |  |  |
| 1. BMI < 18.5 | 2.91 (1.03) | 1.74 | 0.78; 3.89 | 6. BMI < 18.5 | 8.67 (2.37) | 7.21 | 3.58; 14.51 | 6-10 > 2-5 |
| 2. BMI 18.5 - 25 | 1.51 (0.15) | REF | REF | 7. BMI 18.5 - 25 | 11.00 (0.82) | 7.19 | 5.49; 9.43 | 7 > 1 |
| 3. BMI 25 - 30 | 0.90 (0.09) | 0.88 | 0.65; 1.17 | 8. BMI 25 - 30 | 6.38 (0.71) | 5.09 | 3.62; 7.15 |  |
| 4. BMI 30 -35 | 0.98 (0.16) | 0.95 | 0.63; 1.42 | 9. BMI 30 -35 | 7.25 (0.95) | 6.25 | 4.42; 8.83 |  |
| 5. BMI 35 + | 1.04 (0.15) | 1.04 | 0.74;1.46 | 10. BMI 35 + | 5.45 (0.82) | 4.28 | 2.85; 6.41 |  |
| **Other Drug** |  |  |  |  |  |  |  |  |
| 1. BMI < 18.5 | 0.26 (0.26) | 0 | 0; 0 | 6. BMI < 18.5 | 2.35 (1.31) | 0 | 0; 0 | 7, 6 > 2-5 |
| 2. BMI 18.5 - 25 | 0.64 (0.10) | REF | REF | 7. BMI 18.5 - 25 | 3.68 (0.44) | 0 | 0; 0 | 8-10 , 2-5 > 1 |
| 3. BMI 25 - 30 | 0.25 (0.05) | 0.44 | 0.06; 3.33 | 8. BMI 25 - 30 | 2.36 (0.38) | 0.69 | 0.04; 11.43 | 8-10 > 6, 7 |
| 4. BMI 30 -35 | 0.22 (0.04) | 1.19 | 0.27; 5.24 | 9. BMI 30 -35 | 2.25 (0.61) | 1.22 | 0.11; 13.75 |  |
| 5. BMI 35 + | 0.33 (0.12) | 0.65 | 0.08; 5.36 | 10. BMI 35 + | 2.37 (0.69) | 2.5 | 0.22; 28.58 |  |
| **SUD (#)** |  |  |  |  |  |  |  |  |
| 1. BMI < 18.5 | 0.12 (0.02) | 0.85 | 0.60; 1.21 | 6. BMI < 18.5 | 0.34 (0.06) | 2.66 | 1.91; 3.70 | 6-10 > 1-5 |
| 2. BMI 18.5 - 25 | 0.14 (0.00) | REF | REF | 7. BMI 18.5 - 25 | 0.44 (0.02) | 2.64 | 2.44; 2.87 | 2 > 5 |
| 3. BMI 25 - 30 | 0.11 (0.00) | 0.97 | 0.89; 1.06 | 8. BMI 25 - 30 | 0.36 (0.02) | 2.43 | 2.20; 2.67 | 7 > 10 |
| 4. BMI 30 -35 | 0.10 (0.01) | 0.92 | 0.82; 1.04 | 9. BMI 30 -35 | 0.32 (0.02) | 2.28 | 2.01; 2.59 |  |
| 5. BMI 35 + | 0.09 (0.00) | 0.83 | 0.75 | 10. BMI 35 + | 0.27 (0.02) | 1.92 | 1.63; 2.25 |  |

Note: BMI (calculated as kg/m2) ranges correspond to the accepted BMI classification system used by the CDC (CDC, 2020). TUD = tobacco use disorder, aOR = adjusted odds ratio, aRR = adjusted rate ratios, NSC = no significant contrasts, SE = standard error, CI = confidence intervals, M = mean. All groups represented in the contrasts column met the significance threshold which was set at *p*<.05.

Supplemental Table 4. Psychiatric diagnosis with expanded obesity categories

| **MOOD DISORDERS** |  | **No TUD** |  |  |  | **TUD** |  |  |
| --- | --- | --- | --- | --- | --- | --- | --- | --- |
| **MDD** | %(SE) | aOR | 95% CI |  | %(SE) | aOR | 95% CI | Contrasts |
| 1. BMI < 18.5 | 12.45 (2.08) | 1.4 | 0.96; 2.02 | 6. BMI < 18.5 | 14.65 (3.51) | 1.45 | 0.83; 2.53 | 7-10 > 2-4 |
| 2. BMI 18.5 - 25 | 8.8 (0.36) | REF | REF | 7. BMI 18.5 - 25 | 17.19 (1.04) | 2.17 | 1.8; 2.62 | 8-10 > 5 |
| 3. BMI 25 - 30 | 7.54 (0.30) | 1.18 | 1.04; 1.33 | 8. BMI 25 - 30 | 16.37 (0.93) | 2.42 | 2.04; 2.88 | 5, 4 > 2 |
| 4. BMI 30 -35 | 9.29 (0.54) | 1.4 | 1.20; 1.65 | 9. BMI 30 -35 | 17.17 (1.55) | 2.52 | 1.98; 3.21 | 5 > 3 |
| 5. BMI 35 + | 12.39 (0.55) | 1.74 | 1.50; 2.01 | 10. BMI 35 + | 18.91 (1.42) | 2.51 | 2.01; 3.14 |  |
| **Dysthymia** |  |  |  |  |  |  |  |  |
| 1. BMI < 18.5 | 4.00 (1.24) | 1.72 | 0.89; 3.32 | 6. BMI < 18.5 | 2.67 (1.25) | 0.77 | 0.29; 2.04 | 7-10 > 2, 3 |
| 2. BMI 18.5 - 25 | 2.08 (0.18) | REF | REF | 7. BMI 18.5 - 25 | 5.75 (0.611) | 2.23 | 1.70; 2.91 | 9, 10 > 4 |
| 3. BMI 25 - 30 | 2.14 (0.16) | 1.21 | 0.95; 1.53 | 8. BMI 25 - 30 | 4.58 (0.48) | 1.96 | 1.45; 2.64 | 5 > 2, 3 |
| 4. BMI 30 -35 | 2.81 (0.23) | 1.5 | 1.17; 1.92 | 9. BMI 30 -35 | 5.78 (0.83) | 2.55 | 1.80; 3.60 | 4 > 2 |
| 5. BMI 35 + | 4.21 (0.37) | 2.04 | 1.57; 2.65 | 10. BMI 35 + | 6.77 (0.99) | 2.7 | 1.87; 3.90 |  |
| **Bipolar 1 Disorder** |  |  |  |  |  |  |  |  |
| 1. BMI < 18.5 | 0 | 0 | 0; 0 | 6. BMI < 18.5 | 7.88 (3.00) | 9.4 | 3.90; 22.64 | 6-10 > 3, 4 |
| 2. BMI 18.5 - 25 | 0.72 (0.09) | REF | REF | 7. BMI 18.5 - 25 | 3.75 (0.51) | 4.11 | 2.80; 6.03 | 10, 8 > 5 |
| 3. BMI 25 - 30 | 0.71 (0.07) | 1.13 | 0.79; 1.60 | 8. BMI 25 - 30 | 4.05 (0.52) | 4.93 | 3.20; 7.59 | 2-5 > 1 |
| 4. BMI 30 -35 | 0.99 (0.14) | 1.51 | 0.98; 2,30 | 9. BMI 30 -35 | 4.00 (0.69) | 4.89 | 2.87; 8.33 | 5 > 2, 3 |
| 5. BMI 35 + | 1.52 (0.28) | 2.18 | 1.37; 3.48 | 10. BMI 35 + | 6.25 (1.18) | 7.15; | 4.27; 11.96 |  |
| **ANXIETY DISORDERS** |  |  |  |  |  |  |  |  |
| **Panic disorder** |  |  |  |  |  |  |  |  |
| 1. BMI < 18.5 | 1.27 (0.69) | 0.59 | 0.19; 1.79 | 6. BMI < 18.5 | 9.05 (2.70) | 3.37 | 1.70; 6.64 | 7, 10 > 1 |
| 2. BMI 18.5 - 25 | 1.94 (0.16) | REF | REF | 7. BMI 18.5 - 25 | 7.36 (0.60) | 3.25 | 2.51; 4.20 | 6-10 > 2, 3 |
| 3. BMI 25 - 30 | 1.72 (0.15) | 1.13 | 0.86; 1.47 | 8. BMI 25 - 30 | 5.70 (0.59) | 2.88 | 2.17; 3.82 | 7, 8, 10 > 4, 5 |
| 4. BMI 30 -35 | 2.32 (0.27) | 1.39 | 1.05; 1.83 | 9. BMI 30 -35 | 5.09 (0.73) | 2.35 | 1.70; 3.25 | 5 > 2 |
| 5. BMI 35 + | 3.58 (0.39) | 1.83 | 1.36; 2.46 | 10. BMI 35 + | 10.34 (1.34) | 4.47 | 3.16; 6.33 | 10 > 9 |
| **Agoraphobia** |  |  |  |  |  |  |  |  |
| 1. BMI < 18.5 | 1.66 (0.92) | 1.85 | 0.60; 5.71 | 6. BMI < 18.5 | 3.72 (1.44) | 3.24 | 1.38; 7.61 | 7-10 > 2 |
| 2. BMI 18.5 - 25 | 0.81 (0.09) | REF | REF | 7. BMI 18.5 - 25 | 3.44 (0.46) | 3.69 | 2.42; 5.65 | 7, 8, 10 > 3, 4 |
| 3. BMI 25 - 30 | 0.96 (0.10) | 1.5 | 1.05; 2.12 | 8. BMI 25 - 30 | 2.85 (0.43) | 3.51 | 2.27; 5.44 | 5 > 2, 3 |
| 4. BMI 30 -35 | 1.11 (0.15) | 1.59 | 1.11; 2.27 | 9. BMI 30 -35 | 2.45 (0.49) | 2.82 | 1.64; 4.85 |  |
| 5. BMI 35 + | 2.16 (0.32) | 2.69 | 1.77; 4.09 | 10. BMI 35 + | 4.43 (1.24) | 4.52 | 2.38; 8.59 |  |
| **Social Phobia** |  |  |  |  |  |  |  |  |
| 1. BMI < 18.5 | 2.36 (0.93) | 1 | 0.43; 2.33 | 6. BMI < 18.5 | 3.14 (1.40) | 0.96 | 0.38; 2.44 | 7-10 > 2 |
| 2. BMI 18.5 - 25 | 2.19 (0.17) | REF | REF | 7. BMI 18.5 - 25 | 5.26 (0.48) | 1.89 | 1.44; 2.48 | 7, 8, 10 > 3 |
| 3. BMI 25 - 30 | 1.92 (0.19) | 1.02 | 0.80; 1.30 | 8. BMI 25 - 30 | 4.73 (0.54) | 1.94 | 1.48; 2.54 |  |
| 4. BMI 30 -35 | 2.36 (0.24) | 1.3 | 1.02; 1.65 | 9. BMI 30 -35 | 4.65 (0.72) | 1.9 | 1.31; 2.75 |  |
| 5. BMI 35 + | 3.19 (0.35) | 1.45 | 1.10; 1.89 | 10. BMI 35 + | 6.10 (0.82) | 2.27 | 1.66; 3.11 |  |
| **Specific Phobia** |  |  |  |  |  |  |  |  |
| 1. BMI < 18.5 | 4.89 (1.21 | 0.94 | 0.55; 1.62 | 6. BMI < 18.5 | 13.82 (3.88) | 2.61 | 1.35; 5.07 | 7, 8, 10 > 2, 3 |
| 2. BMI 18.5 - 25 | 4.73 (0.28) | REF | REF | 7. BMI 18.5 - 25 | 8.57 (0.61) | 1.81 | 1.49; 2.19 | 5 > 2 |
| 3. BMI 25 - 30 | 4.48 (0.29) | 1.13 | 0.94; 1.34 | 8. BMI 25 - 30 | 7.98 (0.70) | 1.92 | 1.51; 2.43 |  |
| 4. BMI 30 -35 | 5.62 (0.36) | 1.33 | 1.09; 1.63 | 9. BMI 30 -35 | 6.47 (0.73) | 1.44 | 1.10; 1.88 |  |
| 5. BMI 35 + | 6.96 (0.59) | 1.52 | 1.17; 1.96 | 10. BMI 35 + | 9.54 (1.28) | 2.01 | 1.45; 2.78 |  |
| **Generalized Anxiety Disorder** |  |  |  |  |  |  |  |  |
| 1. BMI < 18.5 | 5.44 (1.74) | 1.38 | 0.69; 2.79 | 6. BMI < 18.5 | 14.62 (2.80) | 3.73 | 2.28; 6.12 | 6-10 > 2-4 |
| 2. BMI 18.5 - 25 | 3.69 (0.25) | REF | REF | 7. BMI 18.5 - 25 | 9.59 (0.70) | 2.69 | 2.13; 3.39 | 7, 10 > 5 > 3 |
| 3. BMI 25 - 30 | 3.75 (0.22) | 1.17 | 0.96; 1.42 | 8. BMI 25 - 30 | 8.18 (0.67) | 2.46 | 1.98; 3.07 | 4, 5 > 2 |
| 4. BMI 30 -35 | 4.83 (0.33) | 1.47 | 1.18; 1.83 | 9. BMI 30 -35 | 8.75 (1.07) | 2.57 | 1.84; 3.59 |  |
| 5. BMI 35 + | 6.44 (0.54) | 1.83 | 1.45; 2.31 | 10. BMI 35 + | 12.4 (1.40) | 3.54 | 2.60; 4.82 |  |
| **PTSD** |  |  |  |  |  |  |  |  |
| 1. BMI < 18.5 | 3.48 (1.16) | 1.12 | 0.55; 2.28 | 6. BMI < 18.5 | 13.27 (3.74) | 3.62 | 1.83; 7.17 | 6-10 > 2 |
| 2. BMI 18.5 - 25 | 2.83 (0.18) | REF | REF | 7. BMI 18.5 - 25 | 8.53 (0.71) | 2.6 | 2.07; 3.27 | 7-10 > 3, 4 |
| 3. BMI 25 - 30 | 3.06 (0.21) | 1.31 | 1.09; 1.59 | 8. BMI 25 - 30 | 8.82 (0.72) | 3.14 | 2.45; 4.03 | 8, 9 > 5 > 2, 3 |
| 4. BMI 30 -35 | 3.94 (0.37) | 1.56 | 1.20; 2.01 | 9. BMI 30 -35 | 10.21 (0.99) | 3.51 | 2.71; 4.55 | 4 > 2 |
| 5. BMI 35 + | 5.94 (0.48) | 2.06 | 1.65; 2.58 | 10. BMI 35 + | 11.05 (1.31) | 3.3 | 2.42; 4.51 |  |
| **PERSONALITY DISORDERS** |  |  |  |  |  |  |  |  |
| **Schizotypal Personality Disorder** |  |  |  |  |  |  |  |  |
| 1. BMI < 18.5 | 4.80 (1.25) | 1.7 | 0.94; 3.06 | 6. BMI < 18.5 | 10.02 (2.84) | 2.85 | 1.51; 5.38 | 6-10 > 2 |
| 2. BMI 18.5 - 25 | 2.60 (0.21) | REF | REF | 7. BMI 18.5 - 25 | 10.05 (0.73) | 2.92 | 2.31;3.68 | 7-10 > 3, 4 |
| 3. BMI 25 - 30 | 2.91 (0.20) | 1.26 | 1.01; 1.59 | 8. BMI 25 - 30 | 8.45 (0.74) | 2.66 | 2.07; 3.43 | 7, 8, 10 > 5 > 2 |
| 4. BMI 30 -35 | 3.48 (0.25) | 1.39 | 1.12; 1.71 | 9. BMI 30 -35 | 7.84 (0.93) | 2.46 | 1.78; 3.41 | 4 >2 |
| 5. BMI 35 + | 4.61 (0.38) | 1.68 | 1.30; 2.16 | 10. BMI 35 + | 11.86 (1.47) | 3.53 | 2.50; 4.98 |  |
| **Borderline Personality Disorder** |  |  |  |  |  |  |  |  |
| 1. BMI < 18.5 | 8.11 (1.54) | 1.31 | 0.85; 2.04 | 6. BMI < 18.5 | 26.06 (4.29) | 3.95 | 2.49; 6.27 | 6-10 > 1-5 |
| 2. BMI 18.5 - 25 | 5.85 (0.32) | REF | REF | 7. BMI 18.5 - 25 | 21.86 (1.20) | 3.23 | 2.71; 3.84 | 5 > 2, 3 |
| 3. BMI 25 - 30 | 6.42 (0.30) | 1.23 | 1.06; 1.43 | 8. BMI 25 - 30 | 21.83 (1.12) | 3.56 | 3.03; 4.19 | 4 > 2 |
| 4. BMI 30 -35 | 8.15 (0.51) | 1.48 | 1.25; 1.75 | 9. BMI 30 -35 | 20.79 (1.38) | 3.36 | 2.67; 4.23 |  |
| 5. BMI 35 + | 10.32 (0.60) | 1.76 | 1.47; 2.12 | 10. BMI 35 + | 27.01 (2.17) | 4.32 | 3.44; 5.44 |  |
| **Antisocial Personality Disorder** |  |  |  |  |  |  |  |  |
| 1. BMI < 18.5 | 0.82 (0.60) | 0.71 | 0.15; 3.26 | 6. BMI < 18.5 | 6.14 (2.12) | 3.98 | 1.77; 8.93 | 6-10 > 2-4 |
| 2. BMI 18.5 - 25 | 1.49 (0.16) | REF | REF | 7. BMI 18.5 - 25 | 7.69 (0.76) | 3.63 | 2.72; 4.85 | 7, 8 > 5 |
| 3. BMI 25 - 30 | 1.47 (0.14) | 1.07 | 0.80; 1.42 | 8. BMI 25 - 30 | 6.19 (0.51) | 2.87 | 2.11; 3.91 |  |
| 4. BMI 30 -35 | 1.51 (0.19) | 1.04 | 0.76; 1.43 | 9. BMI 30 -35 | 5.94 (0.79) | 2.82 | 1.87; 4.24 |  |
| 5. BMI 35 + | 2.49 (0.33) | 1.67 | 1.11; 2.53 | 10. BMI 35 + | 7.30 (1.55) | 3.43 | 1.96; 6.00 |  |
| **Mental Health Disorders (#)** |  |  |  |  |  |  |  |  |
| 1. BMI < 18.5 | 0.49 (0.08) | 1.22 | 0.89; 1.67 | 6. BMI < 18.5 | 1.25 (0.155) | 2.46 | 1.92; 3.16 | 6-10 > 1-4 |
| 2. BMI 18.5 - 25 | 0.37 (0.01) | REF | REF | 7. BMI 18.5 - 25 | 1.09 (0.05) | 2.4 | 2.16; 2.66 | 7-10 > 5 |
| 3. BMI 25 - 30 | 0.37 (0.01) | 1.16 | 1.06; 1.27 | 8. BMI 25 - 30 | 0.99 (0.04) | 2.45 | 2.22; 2.70 | 5 > 4 > 3 > 2 |
| 4. BMI 30 -35 | 0.46 (0.02) | 1.37 | 1.25; 1.51 | 9. BMI 30 -35 | 0.99 (0.05) | 2.39 | 2.12; 2.69 |  |
| 5. BMI 35 + | 0.64 (0.03) | 1.69 | 1.52; 1.89 | 10. BMI 35 + | 1.32 (0.09) | 2.88 | 2.47; 3.36 |  |

Note: BMI (calculated as kg/m2) ranges correspond to the accepted BMI classification system used by the CDC (CDC, 2020). TUD = tobacco use disorder, aOR = adjusted odds ratio, aRR = adjusted rate ratios, NSC = no significant contrasts, SE = standard error, CI = confidence intervals, M = mean. All groups represented in the contrasts column met the significance threshold which was set at *p*<.05.
